# Adherence to the Mediterranean-Style Eating Pattern and Macular Degeneration: A Systematic Review of Observational Studies

**DOI:** 10.3390/nu14102028

**Published:** 2022-05-12

**Authors:** Annalisa Gastaldello, Francesca Giampieri, José L. Quiles, María D. Navarro-Hortal, Silvia Aparicio, Eduardo García Villena, Kilian Tutusaus Pifarre, Rachele De Giuseppe, Giuseppe Grosso, Danila Cianciosi, Tamara Y. Forbes-Hernández, Seyed M. Nabavi, Maurizio Battino

**Affiliations:** 1 Research Group on Foods, Nutritional Biochemistry and Health, Universidad Europea del Atlántico, Isabel Torres 21, 39011 Santander, Spain; annalisa80it@yahoo.it (A.G.); jlquiles@ugr.es (J.L.Q.); silvia.aparicio@uneatlantico.es (S.A.); eduardo.garcia@uneatlantico.es (E.G.V.); kilian.tutusaus@uneatlantico.es (K.T.P.); 2 Department of Biochemistry, Faculty of Sciences, King Abdulaziz University, Jeddah 80200, Saudi Arabia; 3 Department of Physiology, Institute of Nutrition and Food Technology “José Mataix”, Biomedical Research Centre, University of Granada, 18100 Granada, Spain; mdnavarro@ugr.es (M.D.N.-H.); tforbes@ugr.es (T.Y.F.-H.); 4 Faculdade de Ciências Sociais e Humanas, Universidade Internacional do Cuanza Bairro Kaluanda, Cuito EN 250, Bié, Angola; 5 Department de Salud, Universidad Internacional Iberoamericana Campeche, Campeche 24560, Mexico; 6 Laboratory of Dietetics and Clinical Nutrition, Department of Public Health, Experimental and Forensic Medicine, University of Pavia, 27100 Pavia, Italy; rachele.degiuseppe@unipv.it; 7 Department of Biomedical and Biotechnological Sciences, University of Catania, 95124 Catania, Italy; giuseppe.grosso@unict.it; 8 Department of Clinical Sciences, Polytechnic University of Marche, 60131 Ancona, Italy; d.cianciosi@pm.univpm.it; 9 Applied Biotechnology Research Center, Baqiyatallah University of Medical Sciences, Tehran 1435916471, Iran; nabavi208@gmail.com; 10 International Joint Research Laboratory of Intelligent Agriculture and Agri-Products Processing, Jiangsu University, Zhenjiang 212013, China

**Keywords:** macular degeneration, retinal disease, eye disease, maculopathy, drusen, Mediterranean diet, plant-based diets, dietary pattern, eating pattern

## Abstract

Age-related macular degeneration (AMD) is a serious degenerative disease affecting the eyes, and is the main cause of severe vision loss among people >55 years of age in developed countries. Its onset and progression have been associated with several genetic and lifestyle factors, with diet appearing to play a pivotal role in the latter. In particular, dietary eating patterns rich in plant foods have been shown to lower the risk of developing the disease, and to decrease the odds of progressing to more advanced stages in individuals already burdened with early AMD. We systematically reviewed the literature to analyse the relationship between the adherence to a Mediterranean diet, a mainly plant-based dietary pattern, and the onset/progression of AMD. Eight human observational studies were analysed. Despite some differences, they consistently indicate that higher adherence to a Mediterranean eating pattern lowers the odds of developing AMD and decreases the risk of progression to more advanced stages of the disease, establishing the way for preventative measures emphasizing dietary patterns rich in plant-foods.

## 1. Introduction

The retina, as a part of the central nervous system, allows the transformation of light signals into images through the engagement of the optic nerve. The macula, a highly pigmented oval-shaped area at the centre of the retina, rich in photoreceptors, is central to this function, being responsible for providing sharp, clear, and straight-ahead vision, and determining most of our colour vision [1]. As a consequence, the preservation of macula health is of great importance, and its deterioration has large repercussions not only on the health status of the entire visual system, but also on a person’s general physical and psychological wellbeing. Unfortunately, one of the main diseases affecting the eye is a disease of the macula. Indeed, age-related macular degeneration (AMD) is the main cause of severe vision loss in older people (>55 years) in Western countries, and owing to an aging global population, its prevalence is expected to rise in the next few decades, with estimates predicting that around 288 million people will be affected worldwide by 2040 [2]. AMD is classified as early, intermediate, or advanced/late according to the retinal pathological alterations present mostly at the macula level, but not necessarily limited to this area [3,4]. The early stages of the disease are characterized by the presence of small drusen, yellow deposits consisting of lipids and proteins, located under the retinal pigment epithelium, whilst in the intermediate forms larger drusen are present, accompanied by pigmentary abnormalities. Advanced AMD has two forms: (a) geographic atrophy (GA) or “dry AMD”, characterized by the loss of photoreceptors, retinal pigment epithelium and choriocapillaris, and (b) neovascular AMD (nvAMD or “wet AMD”) defined by the growth of new blood vessels in the macula, which may lead to leakage of blood and serum into the retina [5]. Both types of advanced AMD result in loss of central vision, severely impacting the lives of affected individuals who experience difficulties in recognizing faces, reading, and performing daily tasks such as cooking, cleaning, and dressing [6]. Furthermore, they often report mood disorders such as depression, owing to the loss of independence and a diminished quality of life [6,7,8]. The prevalence of AMD increases steeply with age; however, this disease is seen as multifactorial, with both nonmodifiable and modifiable risk factors playing a central role in its onset and progression [9]. Among the former, increasing age, female sex, certain genetic polymorphisms, having a family history of AMD, light skin colour, and light iris colour, are associated with an increased risk of AMD [10,11]; among the latter, smoking is the strongest-recognized determinant of a heightened risk of late AMD [12,13], whilst physical activity may be protective against AMD progression by contributing to a denser macular pigment, and so improving retinal health status [14,15]. Diet is another modifiable behavioural factor affecting disease risk, with epidemiological studies showing that dietary patterns rich in zinc or fruits and vegetables are associated with a lower occurrence of AMD [16,17]. Supplementation with a formulation rich in antioxidant compounds (the AREDS/AREDS 2 formulations) is often recommended for early stage AMD [18], whilst treatment is only possible for nvAMD using biologicals that inhibit vascular endothelial growth factor (VEGF), a key mediator of angiogenesis. Anti-VEGF therapy is used to slow the progression of the disease, and its introduction has decreased the legal blindness rate from nvAMD by more than 50% in many countries [18]. However, the responses to this intervention may vary among patients, and the therapy does not prevent the development of atrophy, leading to vision loss in the long term [19,20]. Furthermore, no FDA-approved therapeutic options are available for GA [21]. Therefore, preventive measures become of paramount importance, also in light of the fact that this disease represents a substantial physical and emotional burden for the affected individuals and their families, and an economic one for healthcare systems around the globe [5].

The Mediterranean diet (Med diet) is a predominantly plant-based dietary pattern traditionally found in countries of the Mediterranean basin such as Greece, Spain, and Southern Italy, which centre meals on antioxidant-rich foods such as vegetables, fruits, whole grains, beans, seeds, nuts, and spices. The major source of fat is olive oil, rich in monosaturated fatty acids, whilst fish, rich in polyunsaturated omega 3 fatty acids, is consumed in moderate amounts, and dairy products are ingested with low-to-moderate frequency. On the other hand, red and processed meat is consumed very sparingly, generally only during special occasions [22]. The Med diet is considered one the healthiest diets, and has been extensively studied. Greater adherence to this eating pattern has been shown to reduce general mortality, mortality from cardiovascular disease and from cancer, and to be associated with healthy aging, a lower incidence of neurodegenerative diseases such as Parkinson’s and Alzheimer’s diseases, and of cardiovascular complications, including stroke [23,24,25]. Being a diet centred on healthy food groups, laden with antioxidants such as lutein, zeaxanthin, and vitamin C and E, it is plausible to postulate that its health benefits would extend to the eye, and particularly to the retina, which is a structure highly vulnerable to oxidative damage. This is because of certain metabolic and anatomical characteristics: (i) the high levels of cumulative irradiation, (ii) the high oxygen tension due to high vascularization, and (iii) the abundance of polyunsaturated fatty acids (PUFAs) in the photoreceptors, which are conducive to the formation of high levels of reactive oxygen species and to lipid oxidation [26,27,28]. Whilst the retina possesses an antioxidant system, which include vitamins C and E and the carotenoids lutein and zeaxanthin [26,29,30], protective compounds introduced with diet might strengthen its defensive mechanisms, and protect it more efficiently from other environmental hazards (e.g., sunlight exposure). Whilst supplementation with antioxidants, such as the vitamins C and E and betacarotene, has been shown to reduce the risk of AMD progression by 25% in 5 years in a large American randomised controlled trial (RCT) [31], supplementation with lutein and zeaxanthin was associated with lower odds of progression only in people at the bottom 20% of the dietary intake of these compounds. This suggests that a diet naturally rich in carotenoids, that is plant-based, and in particular green leafy vegetables, might work as an effective first line of defence [32]. Furthermore, a Cochrane systematic review concluded that the abovementioned findings need to be confirmed in other populations before they can be generalized [33], and another systematic review from the same team found that supplementing with the antioxidant compounds vitamin E or betacarotene will not prevent or delay the onset of AMD, with the same probably applying also to vitamin C [34]. Regarding omega-3 PUFAs, a positive effect of these fats on the development and progression of AMD has been shown only in observational studies, and a Cochrane meta-analysis concluded that there is currently no evidence that increasing levels of omega-3 PUFAs in the diet prevents or slows the progression of this disease [35]. 

The concept that a diet in its entirety is much more than the sum of its parts is a relative new concept in nutritional science, a concept which stems from the understanding that foods contain thousands of compounds synergistically interacting to deliver not only nutrition to the body, but bioactive molecules able to modulate the health status of an organism [36]. These synergistic interactions are often only possible when these molecules are left in their natural matrix, and are extremely difficult to replicate inside supplements, so it is important to evaluate whole dietary patterns rather than single food groups or nutrients when studying the effects that diet may have on health and disease. Indeed, this approach is becoming increasingly popular in nutritional epidemiology [37]. The present article aims to systematically review the literature to assess whether the Mediterranean eating pattern, evaluated in its totality, can significantly prevent the development and/or slow the progression of AMD, owing to its high content of plant-derived, antioxidant-rich foods. As we discuss next, there is strong evidence from cohort, cross-sectional, and case-control studies that this diet is indeed associated with a decreased risk of AMD onset and progression, with potentially important repercussions on preventative measures.

## 2. Materials and Methods

### 2.1. Inclusion Criteria and Search Strategy

To systematically review all studies published in peer-reviewed journals evaluating the relationship between the Med diet and AMD, searches were performed on the 13th and 14th of October 2021 using the following platforms: PubMed/MEDLINE (https://pubmed.ncbi.nlm.nih.gov/, accessed on 13 October 2021), Cochrane CENTRAL (https://www.cochranelibrary.com/central, accessed on 13 October 2021), and ScienceDirect (https://www.sciencedirect.com/, accessed on 14 October 2021). The search strategy included keywords in combination as MeSH terms and text words, selected to capture all the relevant literature, and was structured using the appropriate Boolean operators as follows: (“Retinal Diseases”(MeSH) OR “Retina”(MeSH) OR “retinal disease” OR retina OR “macular degeneration” OR maculopathy) AND (“Diet, Vegetarian”(MeSH) OR “Diet, Mediterranean”(MeSH) OR “plant-based diet” OR vegetarian OR Adventist OR “Mediterranean diet” OR “blue zone” OR “dietary lifestyle” OR “dietary pattern”). The query was formatted according to each search engine used, and in particular for ScienceDirect it was subdivided based on the number of allowed Boolean operators (eight). Searches were not restricted by date or language, and additional articles were extracted from reviews retrieved within searches, and from reference lists of selected articles. This step was manually performed by screening the title of articles within reference lists for relevant terms and concepts. Search results were combined, and duplicates removed, using the reference management software “EndNote”. After this stage, only “research papers” were considered further among the articles retrieved from ScienceDirect. This phase was performed by a single author (A.G.), and checked by a second author (F.G.). A list of abstracts was compiled in order to perform a first screening to evaluate whether the articles met the eligibility criteria: (i) human interventional or observation studies, and (ii) evaluation of the relationship between dietary patterns and AMD or macula-related abnormalities. Meta-analyses, systematic reviews, animal and in vitro studies, and case reports, were excluded. The full texts of the short-listed manuscripts were obtained, and a final selection based on more stringent criteria was performed. These criteria included: (i) evaluation of adherence to the Med diet as exposure variable, and (ii) evaluation of the incidence and/or progression of AMD or related macula pathological alterations as outcome in relation to adherence to the Med diet. The full selection process was first carried out by A.G., and then checked by F.G., and followed the guidelines by The PRISMA Group, the widely employed 27-item checklist used to enhance the transparency of systematic reviews. These items comprise all parts of the review, from title, abstract, to introduction, methods, results, discussion, until funding [38]. 

### 2.2. Assessment of Quality

The quality of retrieved studies was assessed according to the Newcastle–Ottawa Scale for case-control and cohort studies, and using a modified version of this scale for cross-sectional studies [39,40]. Both scales evaluate studies based on three areas (selection, comparability, and outcome/exposure) and have a star-based scoring system, but differ regarding the maximum possible score allowed: for the original Newcastle–Ottawa scale this is 9 stars (with a maximum of 4, 2, and 3, respectively, for each section), whilst for the modified version this is 10 stars (with a maximum of 5, 2, and 3, respectively, for each section). Case-control and cohort studies were deemed high quality if they had a score ≥ 7, medium quality with 4–6 stars, and low quality with ≤3 stars [41]. Cross-sectional studies were considered high quality with 8–10 stars, medium quality with 6–7 stars, and low quality with ≤5 stars [42].

## 3. Results

### 3.1. Search Outcomes and Study Quality Assessment

**Figure 1:**
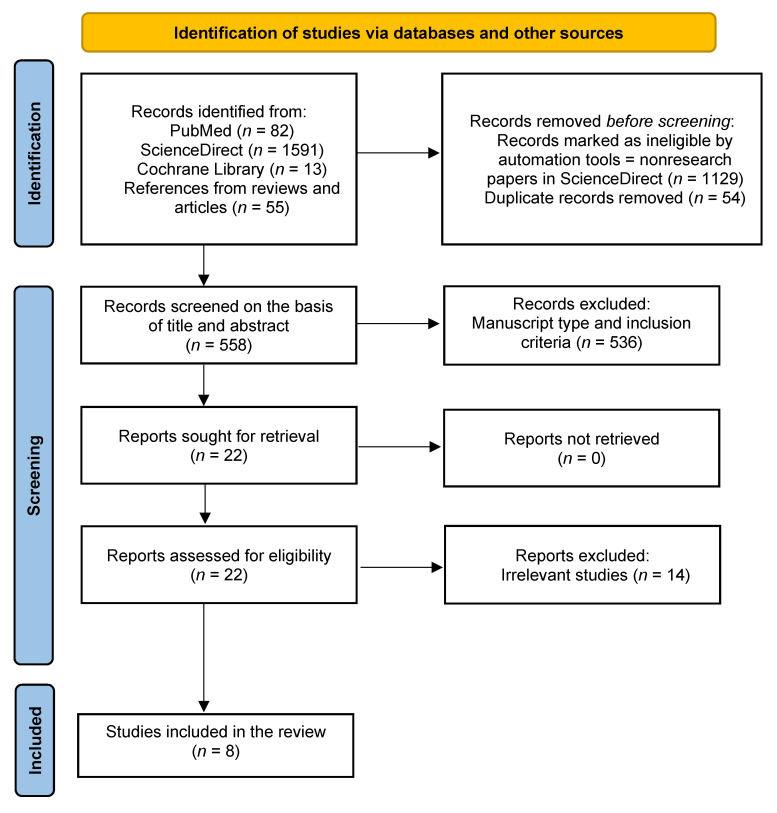
Schematic representation adapted from [43] of the processed followed to identify and select suitable studies according to the PRISMA guidelines.

A flow diagram was created to keep track of the systematic search, as shown in Figure 1. This was adapted from [38,43]. The initial search yielded a total of 1741 potentially relevant articles. The removal of duplicates and nonresearch articles from those retrieved from ScienceDirect, left 558 studies that were screened on the basis of the abstract and the title. The 536 articles that did not meet the inclusion criteria were removed. The full texts of the remaining 22 studies were obtained and assessed for eligibility, applying the more stringent selection standards described above. Of these, 14 were discarded, and 8 were thoroughly analysed and included in this review. A summary of information extracted from these can be found in Table 1, where studies are presented in chronological order. Of the eight manuscripts analysed, one described a retrospective study [44], two had a cross-sectional design [36,45], two were nested case-control studies derived from the same cross-sectional population-based study [46,47], and three were prospective studies [48,49,50].

The assessment of the quality of the studies is reported in Supplementary Tables S1–S3. All cohort studies were deemed of high quality [44,48,49,50], but all of them were given zero stars regarding the representativeness of the exposed cohort, which was considered limited as they all analysed selected groups of participants, and therefore were not representative of the community. They all gained excellent scores in most areas apart from a cohort study included in the article from 2019 by Merle et al. [49]. This manuscript included two cohorts, which were evaluated separately for quality (Supplementary Table S1). The duration of one of these cohorts, the Alienor study, was considered inappropriate (four years) [51]. Of note, all abovementioned studies included robust methods for the assessment of outcome, which was generally performed by expert ophthalmologists using validated procedures such as retinal photographic grading, which is the current gold standard for AMD assessment. All manuscripts, except one [48], clearly reported performing evaluation of exposure through interviews carried out by trained personnel. Both case-controls studies were deemed of high quality [46,47], but lost one star for failure to mention whether the assessment of exposure was performed through interview blinded to case/control status, and another one for not reporting “nonresponse rate”. The two cross-sectional studies [36,45], evaluated by the modified Newcastle–Ottawa Scale, were considered of high quality, gaining the maximum number of stars in each area, apart from the “nonrespondents” section, because of failure to mention the response rate, and the “representativeness of the sample” section for Mares et al. because the participants were represented by a selected subgroup of people from another study, and included only females [36]. Of note, all eight studies were given full scores for “comparability” as they all accounted for the most important confounding factors such as smoking, age, and gender.

### 3.2. Details and Characteristics of the Studies

The assessment of the adherence to a certain diet is done using scoring systems that generally evaluate how a person’s food habits, ascertained through food frequency questionnaires (FFQs), relate to certain standards of consumption of predetermined food groups. The standards and the food groups may vary among scoring systems appraising the same dietary pattern, so similar studies may implement different methodologies. This is the case for the Med diet, for which a number of possible protocols are used. Since one of the key points of the manuscripts analysed in this systematic review is the evaluation of the adherence to such diet, a brief overview of the different scoring systems will be given next. 

The original method for scoring compliance to the Med diet (mediSCORE in the text and Table 1) was devised by Trichopoulou et al. and validated in a Greek population [52,53]. The score is based on the intake of 9 items: vegetables, legumes, fruit and nuts, dairy, cereals, meat and meat products, fish, alcohol, and monounsaturated fatty acids to saturated fatty acids (MUFAs-to-SFAs) ratio. Intakes above the median of the study receive 1 point; all other intakes receive 0 points. Meat and dairy product consumption less than the median receive 1 point. For ethanol, a value of 1 is assigned to men who consume 10–50 g/day and to women who consume 5–25 g/day; possible scores range from 0 to 9.

**Table 1:** Characteristics of the eight studies included in this review evaluating the association between the Mediterranean diet and AMD.

Article Country Study Name and Design	Period of Data Collection Sample Size Age and Sex	Exposure and Outcome Assessments	Outcome and Compared Variables	Adjusted Confounders	OR or HR (95% CI) and *p*-Value	Study Quality	Notes (See Main Text for Further Comments)
› Mares et al. 2011 [36] › USA › CAREDS: cross-sectional nested in WHIOS (prospective)	› CAREDS baseline: 2001–2004; WHIOS baseline: 1994–1998 › 1313 › 55–74 › F	› Validated, semiquantitative FFQ at WHIOS baseline (122 items) › aMED score (0–9) › aMED quartiles: Q1 = 0–1; Q2 = 2–3; Q3 = 4–5; Q4 = 6–9 › Fundus stereoscopic photography › AMD grading based on a modified Wisconsin grading classification	› Early AMD in at least one eye (*n* = 187) › aMED Q4 (*n* = 53) vs. aMED Q1 (*n* = 490)	(a) Model 1: age, pack-years smoked, history of diabetes, AMD, CVD and HRT, and iris colour (b) Model 2: further adjustment for physical activity	› Model 1: OR = 0.34 (0.08–0.98) *p* = 0.046 › Model 2: OR = 0.44 (0.10–1.27) *p* = 0.23	High (8)	› Selected participantshad intakes of lutein plus zeaxanthin that were above the 78th and below the 28th percentiles › aMED Q4: small sample size › Evaluation of the diet using the HEI showed similar results
› Merle et al. 2015 [48] › USA › Prospective cohort within AREDS (RCT)	› 13 years (enrolment 1992–1998) › 2525 › 55–80 at baseline › M and F	› Validated, self-administered, semiquantitative FFQ (90 items) at AREDS baseline › aMED score (0–9) › aMED tertiles: T1 = low (0–3), T2 = medium (4–5), T3 = high (6–9) › Retinal stereoscopic images › AMD grading at baseline based on the CARMS system	› Progression to advanced AMD (*n* = 1028) › aMED T3 (*n* = 676) vs. aMED T1 (*n* = 852)	› Model 1: age, sex, AREDS treatment, AMD grade at baseline, TEI › Model 2: further adjustment for education, smoking history, BMI, supplement use, and 10 genetic variants (SNPs)	› Model 1: HR = 0.74 (0.61–0.90) *p* = 0.005 › Model 2: HR = 0.74 (0.61–0.91) *p* = 0.007	High (7)	› Evaluation of the interaction between aMED score and genetic variations on risk of AMD (10 SNPs analysed in 7 different genes) › Fish and vegetable consumption was associated with lower odds of progression
› Hogg et al. 2017 [45] › Europe (Norway, Estonia, UK, France, Italy, Greece, Spain) › Cross-sectional, within EUREYE study (cross-sectional study with retrospective and current exposure measurements)	› 2001–2002 › 4753 › Mean age = 73.2 ± 5.6 years › M and F	› Semiquantitative FFQ (130 goods) tailored to each country › MDS (0–9) from Martinez-Gonzalez et al. 2004 › MDS score quartiles: Q1 = ≤ 4, Q2 = 5, Q3 = 6, and Q4 = > 6 › Full eye examination and stereoscopic colour fundus digital photography › AMD graded according to the ICS for age-related maculopathy	› Presence of AMD: early (*n* = 2333), large drusen (*n* = 641), GA (*n* = 49), nvAMD (*n* = 109); control (*n* = 2262) › Q4 (*n* = 199) vs. Q1 (*n* = 787)	› Model 1: unadjusted › Model 2: age, sex, country, education, smoking, drinking, history of CVD, aspirin consumption, and diabetes	› Model 1: Early AMD OR = 0.94 (0.85–1.03) *p* = 0.4 Large drusen OR = 0.79 (0.65–0.97) *p* = 0.05 nvAMD OR 0.52 (0.29–0.93) *p* = 0.03 › Model 2: Early AMD OR = 0.96 (0.83–1.11) *p* = 0.9 Large drusen OR = 0.80 (0.65–0.98) *p* = 0.1 nvAMD OR = 0.53 (0.27–1.04) *p* = 0.01	High (9)	› No association between MDS and prevalence of GA
› Nunes et al. 2018 [46] › Portugal › Nested case-control study within the “Epidemiologic Study of the Prevalence of Age-Related Macular Degeneration in Portugal: The Coimbra Eye Study” (cross-sectional) [54]	› 2012–2014 (Coimbra study = 2009–2011) › 1992 › >55 years › M and F	› Validated FFQ (86 items) › mediSCORE (0–9); high adherence = ≥6 › Complete ophthalmological examination and digital mydriatic colour fundus photography › AMD graded according to the ICS for age-related maculopathy (as in Hogg et al. 2016)	› AMD: case group = 768 (control = 1224, age and sex-matched) › High mediSCORE vs. prevalence of AMD	› Age, sex, BMI, abdominal perimeter, physical activity, smoking status, diabetes, and hypertension	› OR = 0.73 (0.58–0.93) *p* = 0.009	High (7)	› Food group analysis: higher consumption of vegetables reduced odds of AMD onset by 36% (OR = 0.63 (0.52–0.76), *p* < 0.001), and higher intake of nuts and fruits lowered odds by 21% (OR = 0.78, (0.65–0.94), *p* = 0.010) › Cases were significantly older
› Raimundo et al. 2018 [47] › Portugal › Nested case-control study within the “Epidemiologic Study of the Prevalence of Age-Related Macular Degeneration in Portugal: The Coimbra Eye Study” (cross-sectional)	› 2012–2014 (Coimbra study = 2009–2011) › 883 › >55 years › M and F	› Same as Nunes et al. 2018	› AMD: case group = 434 (control = 449, age and sex-matched) › High mediSCORE vs. prevalence of AMD	› Age, sex, smoking, calories consumption	› OR = 0.62 (0.38–0.97) *p* = 0.041	High (7)	› Physical activity and fruit consumption were higher in controls (*p* = 0.012 and *p* = 0.029, respectively) › Consumption of 150 g fruit lowered odds by 10% (OR = 0.90 (0.82–0.98; *p* = 0.028)
› Merle et al. 2019 [49] › Europe › Prospective cohort study of the Rotterdam Study I (RS-I) and Antioxydants, Lipides Essentiels, Nutrition et maladies Oculaires (Alienor) study populations, part of the EYE-RISK project	RS-I › 21 years (1990–2011, mean follow-up time 9.9 y) › 4446 › ≥ 55 years › M and F Alienor › 6 years (2006–2012, mean follow-up time 4.1) › 550 › ≥73 years › M and F	› RS-I: 170-item validated semiquantitative FFQ at baseline › Alienor: 40-item validated FFQ at baseline and a 24 h dietary recall › mediSCORE (0–9) › Three groups: low (0–3), medium (4–5), high (6–9) › Ophthalmologic examinations and fundus photographs › AMD graded based on the Wisconsin Age-Related System (RS-I) and the ICS (Alienor)	› Progression to advanced AMD (*n* = 155;RS-I = 117; Alienor = 38) with subtype analysis › mediSCORE high (RS-I *n* = 947; Alienor *n* = 143) vs. mediSCORE low (RS-I, *n* = 1376; Alienor, *n* = 171)	› Model 1: unadjusted › Model 2: age, sex, AMD grade at baseline (no or early AMD), TEI, education, BMI, smoking, multivitamin or mineral supplement use, diabetes, and hypercholesterolemia	› Model 1: RS-I, HR = 0.56 (0.33–0.96) *p* = 0.036; Alienor, ns; Combined, HR = 0.53 (0.33–0.84) *p* = 0.009) › Model 2: RS-I and Alienor alone = ns Combined, HR = 0.59 (0.37–0.95) *p* = 0.04 › No association with nvAMD › GA → RS-I, HR = 0.41 (0.16–1.03) *p* = 0.046; Alienor, ns; Combined, HR = 0.42 (0.20–0.90) *p* = 0.04	High (RS-I = 8; Alienor = 7)	› Association remain after adjustment for two AMD-related SNPs › No single Med diet component was associated with the incidence of advanced AMD
› Keenan et al. 2020 [44] › USA › Retrospective analysis of two RCTs: Age-Related Eye Disease Study (AREDS) and AREDS2	› 13 years (median follow-up 10.2 years), enrolment AREDS 1992–1998; AREDS2 2006–2008 › 7756 (13,204 eyes) › 71 ± 6.6 years › 56.5% F	› AREDS: 90-item, validated, semiquantitative FFQ at baseline AREDS2: 131-item, validated semiquantitative FFQ at baseline › aMED score (modified), ranging from 9 to 36 in main analysis, with assessment using quartile ranks (see main text for details), and from 0 to 9 in sensitivity analyses with assessment using sex-specific medians › Population divided in tertiles: T1 = low, T2 = medium, T3 = high › Eye examinations and colour fundus photographs › AMD graded based on the Wisconsin Age-Related System	› Progression to advanced AMD (AREDS, *n* = 2273; AREDS2, *n* = 2763), with subtype analysis › T3 (AREDS, *n* = 1349; AREDS2, *n* = 1224) and T2 (AREDS, *n* = 1436; AREDS2, *n* = 1101) vs. T1 (AREDS, *n* = 1470; AREDS2, *n* = 1286)	› Treatment assignment, age, sex, smoking, TEI, BMI (for AREDS only), and correlation between eyes › In combined AREDS/AREDS2 analyses, adjustment was also made for the cohort	› Combined cohort: Advanced AMD HRs = T2: 0.87 (0.80–0.94) *p* = 0.001; T3: 0.78 (0.71–0.85) *p* < 0.0001 › Subtypes › GA HRs = T2: 0.80 (0.71–0.90) *p* = 0.0002; T3: 0.71 (0.63–0.80) *p* < 0.0001 › nvAMD HRs = T2: 0.90(0.80–1.01) *p* = 0.08; T3: 0.84 (0.75–0.95) *p* = 0.005 › Large drusen HR = 0.79 (0.68–0.93) *p* = 0.004	High (8)	› Analysis of interaction between aMED and genotype: in AREDS, protective effect was present only in subject with one particular protective allele › Sensitivity analyses: results showed similar pattern but were partially attenuated › Analysis of individual components of the Med diet showed that higher fish consumption was inversely associated with AMD progression
› Merle et al. 2020 [50] › USA › Prospective cohort within AREDS (RCT)	› 13 years (enrolment from 1992 to 1998) › 1838 › 55–80 (at baseline) › M and F	› Validated, self-administered, 90-item, semiquantitative FFQ at baseline › aMED (0–9) › Two groups: low aMED (0–3) or medium-high aMED (4–9) › Complete eye examination and retinal stereoscopic colour images › Maximal drusen size graded in a ordinal scale as detailed in the figure legend	› Drusen size progression (*n* = 587), defined as an eye advancing at least two grades during the study period (from grade 0 to 2, or grade 1 to 3, or grade 2 to 4) › Medium-high aMED vs. low aMED	› Age, sex, education, smoking, BMI, AREDS treatment, multivitamin supplement use, TEI, genetic variants, and maximum drusen size category at baseline in each eye	› HR = 0.83 (0.68–0.99) *p* = 0.049	High (8)	› Drusen = major hallmark of AMD

Characteristics of the eight studies included in this review evaluating the association between the Mediterranean diet and age-related macular degeneration. Abbreviations: AMD: Age-related Macular Degeneration; aMED: alternative or alternate Mediterranean diet score; AREDS/AREDS2: Age-Related Eye Disease Study; BMI: body mass index; CAREDS: Carotenoids in Age-Related Eye Disease Study; CARMS: Clinical Age-Related Maculopathy Staging; CVD: cardiovascular disease; EUREYE: European Eye (study) [55]; FFQ: Food Frequency Questionnaire; GA: geographic atrophy; HR: Hazard Ratio; HEI: Healthy Eating Index; HRT: hormone replacement therapy; ICS: International Classification System; MDS: Mediterranean diet score; mediSCORE: Mediterranean score; ns: not significant; nvAMD: neovascular AMD; OR: Odds Ratio; RCT: randomised controlled trial; SNPs: single nucleotide polymorphisms; TEI: total energy intake; WARMGS: Wisconsin Age-Related Maculopathy Grading System; WHIOS: Women’s Health Initiative Observational Study [56]. Tertile: a statistical value of a data set representing one-third of a given population; quartile: a statistical value of a data set representing 25% of a given population. AMD grading systems employed in the studies: WARMGS: early AMD = absence of signs of advanced AMD and the presence of (1) soft indistinct or reticular drusen or (2) hard distinct or soft distinct drusen with pigmentary abnormalities. Late AMD = presence of either (1) geographic atrophy or (2) exudative AMD. Exudative AMD is defined as the presence of any of the following exudative lesions: pigment epithelial detachment or age-related retinal detachment, subretinal haemorrhage, subretinal scar (subretinal fibrous scar), or prior laser treatment for exudative AMD [57,58]. CARMS (Clinical Age-Related Maculopathy Staging) = no AMD (few small drusen, <63 μm, grade 1); early AMD (drusen within 63–124 μm, grade 2); intermediate AMD (large drusen ≥ 125 μm grade 3); GA (geographic atrophy, both central and noncentral, grade 4); neovascular disease (hemorrhagic retinal detachment, haemorrhage under the retina or retinal pigment epithelium, subretinal fibrosis, grade 5) [48]. ICS: grade 0 = macula free of drusen or pigmentary irregularities or with hard drusen (<63 μm) only; early AMD is subdivided as follows: grade 1 = soft distinct drusen (≥63 μm) or pigmentary abnormalities; grade 2 = soft indistinct drusen (≥125 μm) or reticular drusen only or soft distinct drusen (≥63 μm) with pigmentary abnormalities; grade 3 = soft indistinct drusen (≥125 μm) or reticular drusen with pigmentary abnormalities; advanced AMD = grade 4 = presence of nvAMD (presence of serous or hemorrhagic retinal or retinal pigment epithelial detachment, subretinal neovascular membrane, periretinal fibrous scar) or GA (well-demarcated area of retinal pigment atrophy with visible choroidal vessels). Large drusen (≥125 μm) in any grade of early AMD also is categorized as a separate outcome [59,60]. In Mares et al. [36], the classification was based on a modified WARMGS and was as follows: early AMD = presence of either (1) large drusen (≥1 large drusen (≥125 μm) or extensive intermediate drusen (area ≥ 360 μm when soft indistinct drusen are present or ≥650 μm when soft indistinct drusen are absent)) or (2) pigmentary abnormalities of the retinal pigment epithelium (an increase or decrease in pigmentation accompanied with ≥1 drusen (≥63 μm)). This manuscript only considered early AMD. In Merle et al. [50] the classification was as followed: 0 = no drusen or questionable drusen; 1 = small drusen (<63 μm); 2 = intermediate drusen (63–124 μm); 3 = large drusen (125–249 μm); 4 = very large drusen (≥250 μm).

A modification of this score is represented by the validated “Alternative” or “Alternate” Mediterranean diet score (aMED in the text and Table 1) introduced by Fung at al. to better represent the dietary patterns observed in the US population [54]. In this system, potato products are excluded from the vegetable group, fruit and nuts are in two separate groups, the dairy group is eliminated, the cereals group is substituted by the whole-grains group, the meat group is restricted to red and processed meats, and for alcohol intake 1 point is assigned between 5 and 15 g/day. As in the original version, points are assigned comparing intakes for all groups, except alcohol, with the median of the study participants, and possible scores range from 0 to 9 points.

Another Mediterranean diet score (MDS in the text) was developed by Martinez-Gonzales et al. [57]. The key food items, the number of points assigned, and the set thresholds in this method follow: olive oil (1 point for ≥1 spoon/day), wine (1 point for ≥1 glass/day), fruit (1 point for ≥1 serving/day), vegetables or salad (1 point for ≥1 serving/day), fish (1 point for ≥3 servings/week), legumes (1 point for ≥2 servings/week), meat or meat products (1 point for <1 serving/day). A further point is awarded for a daily serving or more of both fruits and vegetables, and a final point is awarded when consumption of both white bread (<1 serving/day) and rice (<1 serving/week) is low or when consumption of whole-grain bread is high (>5 servings/week). As in the abovementioned systems, possible scores range from 0 to 9 points. Of the eight studies included in this systematic review, three scored the adherence to the Med diet using the original scoring system by Trichopoulou et al. [53]. In the manuscripts by Nunes et al. and by Raimundo et al. [46,47] all points were attributed as described above, whilst in the 2019 article by Merle et al. [49], thresholds differed from the original model for the alcohol group: 1 point was given for consumption equivalent to 1–10 g/day for women and to 5–15 g/day for men, and 0 otherwise. All these studies were performed on European cohorts. The four articles that included American cohorts all employed the aMED score, although applying some modifications compared to the model proposed by Fung et al. [54]. In the manuscript by Mares et al. [36], 1 point is assigned not based on median intakes but as follows: (1) servings of each of the following food components greater than the 75th percentile within the sample: fruits, vegetables, whole grains, legumes, nuts, fish, and the ratio of monounsaturated to saturated fat; (2) less than the 25 percentile for servings of red meat; and (3) alcohol intake of 5 to 25 g/day. Instead, in two of the papers by Merle et al. [48,50], alcohol consumption was evaluated as follows: 1 point to women or men if consumption was within the third quartile of distribution of total alcohol consumption in that population, chosen to represent mild to moderate consumption. In the studies by Keenan et al. [44], the aMED score was modified and adherence level was assessed by quartile ranks (rather than above or below the median) by dividing each food category in quartiles and assigning a score to each quartile, with quartile 4 corresponding to highest intake and therefore highest score (4), except for meat consumption where scoring was reversed (quartile 4 = 1), and for alcohol intake which was converted to a binary format: 4 for intake within the specified intervals and 1 for intake above or below the specified intervals. To calculate the aMedi score for each participant, the quartile values for the 9 components were summed (range 9–36). This method was used to capture more accurately the variation observed in these cohorts. In the same study, the authors also performed sensitivity analyses using the classical aMedi score, as described above. Only one study (Hogg et al. [45]) used the score proposed by Martinez-Gonzales et al. [58].

Early/intermediate and late AMD stages can be documented by retinal colour fundus images, and similarly to the assessment of the adherence to the Med diet, several systems are used to classify AMD both clinically and for research purposes. The grading methods employed in the articles analysed in this review are explained in the legend for Table 1 and consist of: Wisconsin Age-Related Maculopathy Grading System (WARMGS), which is used by two manuscripts [44,49]; the Clinical Age-Related Maculopathy Staging (CARMS), applied by one study [48]; the International Classification System (ICS) employed by three studies [45,46,47]; and a modified version of the WARMGS used by Mares et al. [36]. Furthermore, in the 2020 article by Merle et al. [50] a different grading system is employed, as detailed in the legend of Table 1. These systems differ mostly in their way to classify early/intermediate AMD, and are all based on different combinations of presence, type, size and/or area of drusen, and pigmentary changes. On the other hand, the definition of late AMD is reasonably homogeneous across classification systems, even though some systems such as the CARMS have two separate categories for GA and nvAMD (4 and 5, respectively), whilst the ICS put these two forms of advanced AMD into a single group. The WARMGS has the peculiarity of also taking into consideration the consistency of drusen, dividing them into soft and hard, which is somehow a less objective measure, not taken into consideration by the other systems.

All manuscripts considered for the present systematic review were part of larger studies investigating different aspects of AMD and its association with different behavioural factors, not necessarily including the adherence to the Med diet. To better frame the articles analysed here, some information is given concerning these studies. Three of them, the AREDS (Age-Related Eye Disease Study of the NIH National Eye Institute), the AREDS2, and the CAREDS (Carotenoids in Age-Related Eye Disease Study), represent American cohorts, with AREDS/AREDS2 being RCTs, and CAREDS being an ancillary study of the WHIOS (Women’s Health Initiative Observational Study) [56]. In the AREDS, 4757 participants (55–80 years) were recruited (1992–1998) at 11 US retinal specialty clinics and enrolled into AMD categories (no AMD to unilateral late AMD). The participants were randomly assigned to placebo, antioxidants, zinc, or the combination, and the RCT lasted 5 years. The aim of this study was to assess the effect of antioxidant and mineral supplements on the risk of AMD together with progression to advanced AMD. In AREDS2, 4203 participants (50–85 years) with bilateral large drusen or unilateral late AMD were recruited (2006–2008) at 82 US retinal specialty clinics, and the participants were randomly assigned to receive the supplements that lowered risk of AMD progression in the AREDS (1) alone or with additional (2) lutein/zeaxanthin, (3) docosahexaenoic acid (DHA) plus eicosapentaenoic acid (EPA), or (4) the combination [44]. Similarly, the RCT lasted 5 years. The main purpose of this study was to evaluate the efficacy and safety of the abovementioned supplementation in reducing the risk of developing advanced AMD [44]. The three studies that used data from AREDS/AREDS2 [44,48,50] adjusted their statistical models for the treatments received by the participants. The other American study, the CAREDS, used data collected at WHIOS baseline on dietary and lifestyle habits of the participants on average six years before AMD was assessed [36]. In the WHIOS, 93,676 women were enrolled at forty centres throughout the US (1993–1998), and the aim was to explore the predictors and natural history of important causes of morbidity and mortality in postmenopausal women [59]. The CAREDS women were enrolled among those recruited at three of the forty sites, and were those who had intakes of lutein plus zeaxanthin that were above the 78th and below the 28th percentiles. This choice was made because the intakes of lutein and zeaxanthin significantly correlate with the intake of other healthy foods/nutrients, and this design would maximise statistical power to detect associations with these related aspects of diet [46]. Another study, of which one of the manuscripts analysed here is a part of, is the European Eye (EUREYE) study, which enrolled 4753 participants older than 65 years from seven countries across Europe with widely differing cultures and dietary patterns between 2001 and 2002 [45,55]. It was developed to provide estimates of the prevalence of AMD and to examine associations with lifestyle and environmental factors. Two manuscripts [46,47] were nested in the Coimbra eye study, a cross-sectional population-based study of the prevalence of AMD in Portugal, which recruited 6000 participants over 55 years of age from primary healthcare units in two locations in the centre of Portugal, one in the coastal area (Mira) and the other 70 km away from the sea (Lousã), from 2009 to 2011. A single article analysed two cohorts part of the EYE-RISK project (2015–2019), a large study aiming to identify risk factors, molecular mechanisms, and therapeutic approaches for AMD using data from sixteen European epidemiological cohorts in six countries (Germany, The Netherlands, Spain, France, UK, Switzerland) [49].

Although the aims of this systematic review do not include the evaluation of the interactions between the Med diet and certain genetic polymorphisms associated with AMD, it is worth mentioning that four of the studies analysed here investigated this relationship. It is known that certain genetic variants either increase or decrease the risk of developing AMD. Among the genes involved, there are the following seven: complement factor H (CFH), age-related maculopathy susceptibility 2/high-temperature requirement A serine peptidase 1 (ARMS2/HTRA1), complement component 2 (C2), complement factor B (CFB), complement component 3 (C3), collagen type VIII a 1 (COL8A1), and RAD51 paralog B (RAD51B). These were analysed in the study by Merle et al. [48] in which a high aMED diet score was associated with lower odds of developing advanced AMD in subjects carrying a particular polymorphism of the CFH gene (CFH Y402H) considered nonrisk, whist this association was not present in subjects who were homozygous for a risk allele. This was the first study investigating whether genetic susceptibility could modify the association between the entire diet (rather than specific nutrients) and AMD. Hogg et al. [45] instead found that the relationship between MDS score and the risk of developing AMD was not influenced by the Y204H allele. A later article by Merle et al. investigating two different cohorts from the EYE-RISK consortium also found no association between CFH Y402H and the mediSCORE, and this was true for another gene, the ARMS2 [49]. In the manuscript by Keenan et al. [44], the interaction between the aMED and two single nucleotide polymorphisms (SNPs) at two loci with the highest attributable risk to late AMD, ARMS2 rs10490924 and CFH rs10922109, were analysed. In AREDS, higher aMED was associated with decreased risk of late AMD only in participants with CFH protective alleles, whereas no significant association was found in AREDS2, along with with ARMS2 alleles in both studies.

### 3.3. Incidence and Prevalence of AMD and the Mediterranean Diet

Four of the manuscripts included in this systematic review investigated AMD incidence or prevalence in relation to the adherence to the Med diet [36,45,46,47]. In the article by Mares et al., the incidence of AMD over a period of 6 years was lower in people with higher aMED score (−66%). Importantly though, this association was no longer significant after adjustment for physical activity, suggesting that the effect of the Med diet was not totally independent from this other lifestyle factor. It is worth noting that the number of participants with a high aMED score (Q4 = 6–9) was small (*n* = 53) in comparison with those in Q1 (*n* = 490) (Table 1). Since the comparison was made between Q4 and Q1, this might have had an effect on the results. In addition, Mares et al. also investigated the relation between whole diet and the risk of early AMD using the Healthy Eating Index (HEI), showing that CAREDS participants with the highest quintile of the HEI had lower odds of early AMD (−46%). In the article by Hogg et al., where prevalence of AMD was stratified by AMD stages, higher adherence to the Med diet decreased the odds of developing nvAMD in unadjusted and confounder-adjusted analysis by 48% and 47%, respectively, but did not have significant effects on other forms of the disease (Table 1). In the two case-control studies by Nunes et al. and by Raimundo et al., a high mediSCORE was associated with a lower prevalence of AMD (−27% and −38%, respectively). These investigations also analysed the different food groups of the Med diet and found that high intake of vegetables, nuts, and fruit drove the association between the mediSCORE and AMD, decreasing the odds by 10% to 36% compared to lower consumption (Table 1).

### 3.4. Progression of AMD and Related Retinal Abnormalities and the Mediterranean Diet

The other four articles analysed here investigated the influence of the Med diet on disease progression [44,48,49,50], including one by Merle et al. from 2020 specifically studying the progression of drusen size rather than AMD per se, drusen being an hallmark of AMD [50]. In the article by Merle et al. from 2015 [48], the authors demonstrated that higher aMED score decreased the risk of progression of AMD by 36% (aMED T3 vs. aMED T1, Table 1). Additionally, food group analyses showed that higher vegetables and fish consumption lowered the odds of progression by 15% and 14%, respectively, in the adjusted model. In a subsequent study by Merle et al. considering two European cohorts, the Rotterdam Study I (RS-I) and the Antioxydants, Lipides Essentiels, Nutrition et maladies Oculaires (Alienor) study [50,51], the risk of progression to advance AMD was diminished by high mediSCORE compared to low only in the RS-I and in the combined analysis (RS-I and Alienor study together, −44% and −47%, respectively, Table 1), but not in the Alienor study alone, in the unadjusted model. In the adjusted model, the odds were reduced only in the combined analysis (–41%). AMD subgroup analysis showed that there was no association between mediSCORE and nvAMD, whilst the risk was lowered for GA only in RS-I and combined analysis, but not in the Alienor study alone (−69% and 68%, respectively, Table 1). Furthermore, the inverse association between the adherence to the Med diet and AMD progression remained after adjustment for two SNPs (see above). These results are somehow in contrast with the results by Hogg et al. [45], which showed that higher adherence to the Med diet was associated with lower odds of nvAMD but not GA. Analysis of the interaction between the single food groups of the mediSCORE and AMD progression did not reveal any association. Similar results were instead reported by Keenan et al. in two American cohorts, the AREDS and the AREDS2 [44]. Retrospective analysis of these two studies showed that higher modified aMED score (assessed as described earlier) was associated with lower odds of AMD progression (−13% aMED T2 vs. T1, and −22% aMED T3 vs. T1; Table 1) in combined cohorts, with subtype analysis revealing a decreased risk for GA (−20% and 29%, respectively). Higher adherence to the Med diet was associated with a lower risk of nvAMD only when comparison was made between the highest tertile versus the lowest (T3 vs. T1, −16%). It is important to note that the article by Keenan et al. used strong statistical methods. Indeed, they stated that the proportional hazards assumption pertaining to the multivariate proportional hazards regression was tested in all cases, and when not met (in only one case), stratified proportional hazards regression was performed instead. In addition, they performed sensitivity analyses regarding the relationship between the aMED score, which they modified compared to the original one (see above), and the risk of AMD progression by swapping their score for the classical aMED score. This resulted in similar outcomes, although the patterns of association were partially attenuated, and according to the authors this might have been due to loss of information caused by binary assignment above or below the median using the original aMED score [44]. Keenan et al. also analysed Med diet food groups in relation to AMD and showed that higher fish consumption (Q4 vs. Q1) in AREDS diminished the risk of AMD progression by 31%. Furthermore, progression to large drusen, a risk factor for advanced AMD, was evaluated in separate analyses, which indicated that higher aMED (T3 vs. T1) decreased the odds by 21%. In the paper by Merle et al. from 2020 [50], the Med diet is evaluated in relation to drusen size progression rather than AMD progression. We decided to include this article in our systematic review because drusen are the major hallmark of AMD, and even though information are scarce about the factors influencing their progression, it is known that early AMD presenting drusen increases the risk of developing more advance disease [50,61,62]. The definition of drusen progression is reported in the legend of Table 1. In this prospective study within the AREDS, a medium/high adherence to the Med diet as assessed by the aMED lowered the risk of drusen progression by 17% compared to low adherence (score 4–9 vs. 0–3, Table 1).

## 4. Discussion

In this systematic review, eight articles investigating the relationship between the adherence to the Mediterranean eating pattern and onset/progression of AMD were analysed. These studies included a total of 26,056 people, and analysed more than 11,327 outcomes, represented either by new AMD cases or cases in which the disease progressed from earlier to later stages. They demonstrated that higher adherence to the Med diet was associated with a decreased risk of developing AMD, and with lowered odds of progression to more advanced forms. Since similar associations found in studies with different populations support a robust link between a certain outcome and the chosen exposure, our review shows a clear beneficial effect of a predominantly plant-based eating pattern on AMD risk, both from a preventative and an interventional point of view. The pathogenesis of AMD is complex, being multifactorial, and involves several mechanisms such as endothelial dysfunction, inflammation, and oxidative stress, processes common in other degenerative disease [63]. For this reason, the exploration of how a diet centred on foods rich in antioxidants and anti-inflammatory molecules, such as the Med diet, affect AMD onset and development is justified by biological plausibility. Despite the high quality of the studies, some limitations are present. Firstly, as demonstrated by Keenan et al. with their sensitivity analysis [44], the binary system used for assigning points in the assessment of the adherence to the Med diet can be limiting, and a more stratified version could better represent a complex behaviour such as eating, and highlight subtle differences that might have an impact on disease outcomes. Furthermore, different studies adjusted their model for different variables, which might increase the risk of residual confounding, even though all of them made adjustments for at least age, sex (except for Mares et al. in which participants consisted of females only), and smoking, which are the most important confounders. In addition, two out of eight studies were cross-sectional [36,45], and two were case-control studies [46,47], which are more prone to recall and selection biases. It also is worth remembering the genetics play a role in AMD pathogenesis, and that interactions between certain SNPs and diet might play a large role in determining disease onset and/or progression, as shown by some of the studies analysed here.

Nonetheless, the result of our systematic review is important because there are currently no efficient treatments for curing AMD, most notably the GA form, and preventative measures for delaying its onset, in association with interventional strategies aimed at slowing its advancement, are of pivotal significance. This is also true in light of the fact that in recent years, diets emphasising the consumption of plant-derived food groups, and advocating for the reduction in animal-based products, the so-called flexitarian diets, have become more popular. Consequently, a greater number of people might be adhering, or will adhere, to “newer” dietary patterns similar to the more established Med diet, with consequences that are currently unknown concerning general eye health, and particularly AMD risk. This may warrant the execution of future studies similar to those presented here, in order to assess the association between these increasingly widespread eating behaviours and a disease with a profound effect on the physical and psychological wellbeing of people.

To the best of our knowledge, this is the first systematic review evaluating the relationship between the Med diet, a dietary pattern extensively studied for its potential beneficial health effects owing to its high content of plant foods, and AMD. Here, through a thorough selection of the literature, we identified high quality studies demonstrating that higher adherence to the Mediterranean-style eating pattern can delay the onset and progression of AMD. Although further studies might be needed, our findings may encourage the development and implementation of lifestyle-modifying strategies aimed at reducing the risk of AMD onset and progression.

## Data Availability

Not applicable.

## Supplementary Materials

The following supporting information can be downloaded at: https://www.mdpi.com/article/10.3390/nu14102028/s1, Supplementary Table S1. Newcastle–Ottawa Quality Assessment Scale for cohort studies included in the systematic review. Supplementary Table S2. Newcastle–Ottawa Quality Assessment Scale for case-control studies included in the systematic review. Supplementary Table S3. Modified Newcastle–Ottawa Quality Assessment Scale for cross-sectional studies included in the systematic review.
