# Supplementary material for: Adherence to the Mediterranean-Style Eating Pattern and Macular Degeneration: A Systematic Review of Observational Studies
Source: Nutrients. 2022 May 12;14(10):2028. doi: 10.3390/nu14102028 (PMC9144566; doi:10.3390/nu14102028)
Supplement: Supplementary file 1 [file nutrients-14-02028-s001.zip › nutrients-1703284-supplementary.pdf]

Supplementary Table S1. Newcastle-Ottawa Quality Assessment Scale for cohort studies included in the systematic review.

ARTICLE	SELECTION				COMPARABILITY	OUTCOME			TOTAL SCORE	QUALITY
	**Representativeness of the exposed cohort**	**Selection of the non-exposed cohort**	**Ascertainment of exposure**	Demonstration that outcome of interest was not present at start of study		Assessment of outcome	Was follow-up long enough for outcomes to occur	Adequacy of follow up of cohorts		
Merle et al., 2015 [48]		*		*	**	*	*	*	7	High
Merle et al., 2019 [49] RS-I		*	*	*	**	*	*	*	8	High
Alienor		*	*	*	**	*		*	7	High
Keenan et al., 2020 [44]		*	*	*	**	*	*	*	8	High
Merle et al, 2020 [50]		*	*	*	**	*	*	*	8	High

* = this symbol represents the number of stars given to each category according to the star-based scoring systems employed to assess the quality of each study as detailed in the section "Assessment of Quality" in the main text.

Supplementary Table S2. Newcastle-Ottawa Quality Assessment Scale for case-control studies included in the systematic review

ARTICLE	SELECTION				COMPARABILITY	EXPOSURE			TOTAL SCORE	QUALITY
	**Is the case definition adequate?**	**Representativeness of the cases**	Selection of controls	Definition of controls		Ascertainment of exposure	Same method of ascertainment for case and controls	Non-Response rate		
Nunes et al., 2018 [46]	*	*	*	*	**		*		7	High
Raimundo et al., 2018 [47]	*	*	*	*	**		*		7	High

* = this symbol represents the number of stars given to each category according to the star-based scoring systems employed to assess the quality of each study as detailed in the section "Assessment of Quality" in the main text.

[Type text]

Supplementary Table S3. Modified Newcastle-Ottawa Quality Assessment Scale for cross-sectional studies included in the systematic review

ARTICLE	SELECTION				COMPARABILITY	OUTCOME		TOTAL SCORE	QUALITY
	**Representativeness of the sample**	**Sample size**	Non-responders	Ascertainment of the exposure (risk factor)		Assessment of outcome	Statistical test		
Mares et al., 2011[36]		*		**	**	**	*	8	High
Hogg et al., 2016 [45]	*	*		**	**	**	*	9	High

* = this symbol represents the number of stars given to each category according to the star-based scoring systems employed to assess the quality of each study as detailed in the section "Assessment of Quality" in the main text.
